# Medical Items and News of Interest to the Physician

**Published:** 1876-01

**Authors:** 


### Medical Items and News,

### For the Use of Physicians.

CULLED FROM THE STANDARD MEDICAL JOURNALS OF THE WORLD.

NOTE.—These formulæ are intended only for the regular practitioner of medicine, and should be employed only by him, or under his immediate supervision.

**Hysteria in the Male.**

Compression of the testes is recommended by Dr. Foet, who reports one very severe case thus relieved "in less than a minute."

**Dysentery.**

Nitrate of soda, in the dose of from three to six drachms, during the twenty-four hours, where the disease is entirely rectal, is recommended by the *Phila. Med. & Surg. Reporter.*

**Pruritis of Variola.**

To allay the pruritis which accompanies the eruption of variola, and so prevent the patient from lacerating the skin by scratching, Dr. Noel Gueneau de Mussey recommends the following ointment: Bromide of potassium, three grammes; camphor, thirty centigrammes; cerate, thirty grammes. To be applied several times daily.

**Cerebral Rheumatism.**

Three cases of this formidable disease, treated by M. Bouchut, with hydrate of chloral, have recovered. He gave from three to six grammes, (45 to 90 grains), in divided doses, repeated at frequent intervals, until the excitement abated.—*Phila. Med. & Surg. Reporter.*

**Scirrhus of the Breast.**

After the removal of a scirrhus breast in St. Luke's Hospital, the patient suffered severely from pain in and about the wound, which was markedly relieved by the application of cloths wet in a solution of boracic acid.

Pieces of muslin are dipped in a saturated solution of this drug, and then dried. Before being applied they are dipped in water.

**Eczema Rubrum.**

When this disease follows facial erysipelas, tincture of iodine is to be painted over the entire surface. As a result, the disease will be in every way aggravated for one or two days, but when the artificial irritation subsides, the change produced in the tissues by the iodine permits the case to go on to rapid and complete recovery.

**Intermittent Fever**

The following is commended as an excellent preventive for intermittent fever:—

℞
Cincho-Quinine, 45 grs.
Leptandrin, 15 grs.
Xanthoxylin 8 grs. M.

Ft. pilulæ xxx. Sig. One, night and morning.

**Anti-Catarrhal Pills.**

We find this recipe in the *Philadelphia Medical Times*:—

Gum ammoniac, gr. xxx.
Ammonii carbonat, gr. xv.
Ipecac. pulv., gr. iij.
Morphiæ muriat, gr. iss.
Mucilaginis, q. s. M.

Ft. in pil. no. x. Coat with balsam of tolu dissolved in chloroform. Take one pill morning and evening in chronic bronchitis.

**Passage of Pins, etc., per Anum.**

James L. Fite, of Lebanon, Tenn., (*Nashville Jour. Med. & Surg.*), reports the case of a child, three months old, who passed per anum, without difficulty, within two days, twenty-eight pins, two needles, and an agate button. So far as he could discover, the baby had never a pain from the effects of the pins, etc. It was thought that they were given to the baby by a little servant girl on the morning of the day the first pins came away.

**Belgian Medical Laws.**

A judgment has recently been confirmed by the Court of Appeals at Brussels, which was given against Dr. Campenbout, for supplying his patients during three years with homœopathic medicines. A law of 1818 appears to have been the basis of this confirmation, by which a medical practitioner is forbidden to practice pharmacy together with medicine, except in certain cases, although he possesses the diploma of Doctor of Pharmacy; neither is he permitted, even gratuitously, to supply medicines.

**Cardiac Œdema.**

In a lecture at La Charite, on disease of the heart, Professor See advocated the following treatment of œdema and anasarca due to heart affections:—Extract of squills, fifteen grains; powder of squills, ten grains; for ten pills. From six to ten of these pills to be taken daily. At the same time about one drachm of bromide of potassium is administered daily. Under the influence of these two drugs the dropsy is stated to diminish rapidly.

**Nickelization.**

In Plazanet's process a bath is used of 87.5 grammes of sulphate of nickel, 20 of sulphate of ammonia, 17.5 of citric acid, and 2 litres of water. A bath much used in France is formed of a solution of 4 parts of nitrate of nickel in 4 of liquid ammonia, and 150 of water in which 50 parts of sulphate of soda have been dissolved. Using a moderately weak current, the operation is at an end in a few minutes. There is no need to interrupt it by taking the objects out and brushing them. When the film of nickel is of sufficient thickness, the objects are withdrawn from the bath and dried with sawdust.

---

**Pityriasis Capatis.**

Dr. Malassez recommends the following ointment to be thoroughly rubbed into the scalp morning and evening:

℞
Butter of cacao.
Castor oil.
Oil of sweet almonds, āā ℥j.
Turpeth mineral, grs. xv. M.

The hair should be cut short, and the head washed with an alkaline soap every other day.—*Phila. Med. & Surg. Reporter.*

---

**Sore Throat.**

For diphtheritic scarlatina, and other forms of sore throat, Dr. Holden recommends the following:

℞

Corticis peruvianæ flav., ℨ ij.

Acaciæ pulv., ℨ j.

Sacch alb., ℨ ss. M.

S. Mix one-half of this powder in a tablespoonful of cream and apply frequently with a camel-hair brush.

---

**Pruritis.**

Dr. L. Duncan Bulkley's prescriptions for the intolerable pruritis common in fall and winter, are given in the *Transactions of the American Medical Association*, as follows:

℞
Pulv. gum camphor.
Chloral hydrate, āā ℨ j.

Grind well together in a mortar, till they form a fluid, and add slowly, simple cerate, one ounce.

LIQUOR PICIS ALKALINUS.

℞

Potass. causticæ, ℨ j.

Picis liquidæ, ℨ ij.

Aquæ, ℥ v. M.

Dissolve the caustic potass. in the water, and add gradually the tar, mixing them well in a mortar. Use in solution with from 8 to 16 parts of water.

**Traumatic Erysipelas.**

DR. HIRSCHBERG relates the case of a slaughterman who accidentally cut his upper arm, the wound being about five inches long, and penetrating the muscles. A good deal of venous hemorrhage followed. On the second day a bad attack of erysipelas of the arm came on. A two-per-cent. solution of carbolic acid was injected subcutaneously at the upper boundary of the erysipelas, and next day all traces of erysipelas had disappeared. The injections were repeated five times within the next three days, and the wound healed far more rapidly and with much less suppuration than could have been expected.—*Berlin Woch.*

---

**Diphtheria.**

Dr. Wagner, of Fribourg, reports fifteen serious cases of diphtheria cured by the following method. In children too young to gargle, salicylic acid is given in water or wine, in doses of ten to thirty centigrammes, (about 1½ to 4¾ grains), every two hours. For those who are older he prescribes the following gargle:

Salicylic acid, 150 parts.
Alcohol, (to dissolve it), 15 parts.
Distilled water, 150 parts.

To be used every two hours. If the solution deposits any crystals, he dissolves them by warming it.

---

**Fish Diet.**

Dr. A. D. Binkard writes to the *Philadelphia Med. & Sur. Reporter*, as follows: The chief objection to salt fish as an article of diet is its universal fishiness. No one wishes to be constantly reminded of what he has eaten. That which goes to supply our temporal wants should be like alms given to the poor. It should be heard of no more.

It is not generally known that salt mackerel, (*Scomber vulgaris*), and indeed any salt fish, may be eaten with impunity when properly cooked and served up with lemon. A fresh lemon, sliced and laid over a dish of properly cooked salt mackerel, when brought to the table emits an odor when mingled with the fishy odor, that is to most persons absolutely agreeable, and in many persons even whets the appetite.

If the juice of the lemon be now pressed out on to the fish the taste is very much improved. No eructations follow, though you drink freely of water after the meal.

As a vehicle for taking cod-liver oil, lemon juice serves an excellent purpose, and should be especially recommended to patients of a rheumatic diathesis.

**Biliary Calculi Extracted Through the Abdominal Walls.**

M. Brousson, of Nimes, describes the following case: A patient was suffering from a painful, ill-defined tumor, situated in the right flank, between the spine of the ileum and the umbilicus. The tumor was taken to be a suppurating ovary, and was opened with the caustic Vienna-paste. The tumor was found to be due to an accumulation of biliary calculi, of which no less than forty were removed. In less than three months the patient had recovered.—*Journal de Medecine.*

---

**Pneumonia.**

If a patient is seen when the introductory chill is present, and there is a feeble heart action, as is usually the case in those who are addicted to the use of alcoholic drinks, at once give a drachm or half a drachm of chloroform in brandy, or if chloroform is objected to, co. spt. of ether, in from two to four drachm doses. This is recommended upon the ground that something is required which shall stimulate the heart's action, and the above potion is regarded as the most powerful cardiac stimulant at our command.—*N. Y. Hosp. Rep.*

---

**Parisian Police Lanterns.**

Safety lamps of an original construction are used by the night policemen and watchmen of Paris.—A small glass vial holds a piece of phosphorus as large as a pea, upon which is poured boiling olive oil sufficient to fill up about a third of the vial. The latter is then closely stopped by a cork. In use, the stopper is released for a moment, so as to permit the entrance of air to the phosphorus.—The vacant inner space is thereupon lit up, diffusing a clear, and, of course, perfectly harmless light. When the light fades it may be revived by a fresh uncorking. A lamp so prepared will hold good for six months without renewal.

---

**Mucous Polypus.**

Dr. Meplaine, of Moulins, gives the history of a man, thirty years of age, who had been afflicted for a month with mucous polypus of the velum palati. The tumor was growing rapidly, and impeded deglutition and speech, and gave rise to considerable hemorrhage. Applications of chromic and carbolic acids, removals with scissors, &c., afforded no relief, the tumor being constantly reproduced. A drop of acetic acid was injected into the polypus, and it immediately began to shrink; a second injection completed the cure, and five months later there were no symptoms of a return of the disease.—*N. Y. Med. Jour.*

**Burns.**

There has been in hospital for many months a case of extensive burn, in which different applications have been tried. Every new dressing succeeded well for a time, but soon it ceased to prove of advantage. The last agent that has been used, and is used at present, is salicylic acid. The effect is more beneficial than that obtained by any of the former remedies. The method of using it is to form an emulsion with olive oil, one part of the salicylic acid to sixteen parts of oil. This mixture is painted over the ulcerated surface once or twice a day. It gives rise to a slight smarting sensation when first applied, but that soon passes off. *Med. Times.*

---

**Wet Strapping.**

With the object in view of permitting the plaster to remain as long as possible without change when strapping old ulcers, especially such as are of specific nature, found upon the lower extremities, several cases have been dressed in St. Luke's Hospital with what have been called wet straps. These are prepared by passing strips of adhesive plaster through hot carbolized water instead of heating them in the usual manner. The plasters did not get loose as quick as when heated over a spirit lamp, and the ulcers are reported as healing more quickly under this plan of treatment than any which had been adopted.

---

**Too Much Tobacco.**

Prof. Chevalier reports the case of a young man who had laid a wager that he would smoke twelve cigars. He felt decidedly uncomfortable at the end of the eighth, and when he had finished the ninth he was attacked by giddiness and shiverings. These symptoms became worse after the tenth. He refused to leave off smoking, but went home in charge of some friends. He was there attacked by severe pain, and a medical man was called in, who could not, however, stop the progress of the attack, and the patient died in the night.

In the British medical naval report just issued, a fatal case of poisoning by tobacco is mentioned. A boy on the *Implacable* had frequently been reproved for chewing tobacco, and on several occasions swallowed pieces to prevent detection. On the night of his death he was heard breathing stertorously, and efforts to arouse him being in vain, he was taken to the sick bay. His pupils were insensible to the light, and his pulse beat feebly. He died in two or three minutes after. Two small pieces of tobacco were found in his stomach.

**Inunction.**

Dr. Fisher, in the *N. Y. Medical Record*, insists upon the value of this measure for other than mere local purposes. It should be preceded by a warm bath, after which the patient should be clothed in a loose flannel wrapper, long enough to cover the feet and preserve the bed-clothes from soiling. The oil, slightly warmed, should then be liberally applied by the bare hand of the attendant, over the entire body and limbs. The first result is the production of a general sense of comfort, any nervous irritation that may have been present undergoing diminution. A gentle perspiration soon shows itself, sometimes accompanied by an erythmatous eruption, which is, however, but slight and temporary. The general conclusions of the paper are, 1st. That inunction is a valuable method of introducing nutritive material into the body. 2d. The vegetable oils are better suited for inunction than the animal oils or fats. 3d. Inunction is chiefly useful in diseases and derangements in which wasting is a marked symptom, and is especially beneficial in scrofula, syphilis and rickets. It is serviceable in nervous affections, in convalesence from acute disease, in many chronic diseases, especially those of the chest and abdomen, and in children.—*Phila. Med. & Surg. Reporter.*

---

**Chorea.**

Two cases of this disease were seen which presented some points of interest. One patient, a girl æt. 17, was suffering from her second attack; the first attack occurred when she was eleven years of age, was probably developed by over-work, and lasted for one month. Her father died of heart-disease, but her mother is still living and healthy. The treatment since admission had consisted in the use of a general tonic course, and to this arsenic had been added, but without causing any improvement. Strychnia was substituted, beginning with $\frac{1}{50}$ of a grain once a day, and was increased daily until a trifling amount of muscular rigidity was developed in some part of the body. Her improvement was manifest at once and the case went on to recovery.

The second case was a girl æt. 10 years, who had had an attack when she was five years of age, and the attacks had been repeated *yearly*, but had not as a rule lasted very long. Father healthy, and mother died of consumption. Her treatment was to consist of a generous diet and the use of strychnia. There was *no history of rheumatism* in either case. In the latter, the disease was probably due to fright. At the end of three weeks the girl had nearly recovered.—*N. Y. Hosp. Rep.*

**Cod Liver Oil Formula.**

The following formula, by Dr. E. C. Mann, contains phosphoric acid, the use of which will often overcome the nervous exhaustion and depression under which a patient is laboring, and at the same time assist the appetite and digestion:

℞

Yolk of three eggs.

Oleum morrhuæ, ℥ viij.

Sherry wine, ℥ iv.

Acidi phosphorici, } āā ℥j.

Syrupi simplicis, }

Aquæ amygdalæ amaræ, ℥ viij.

Spiriti vini rect., ʒ j. M.

Rub the eggs up in a mortar, adding the oil spoonful by spoonful. Last of all the phosphoric acid.

---

**Insufflation in Intestinal Obstruction.**

Dr. Brun, in a communication to the *Journal De Medicine*, of October, describes a case of intestinal obstruction successfully relieved by means of J. Wood's method of insufflation of air. An œsophageal sound was introduced into the rectum and to the external extremity, the nozzle of an ordinary bellows was attached. Air was then injected till the abdomen had increased materially in volume. The first attempt, however, was unsuccessful. It was repeated and the insufflation continued until considerable abdominial tension was produced and respiration was rendered difficult.—In the course of an hour dejections began and the patient recovered.

---

**Bronchial Catarrh and Winter Cough.**

In a note sent to the *British Medical Journal*, Drs. Sidney Ringer and Wm. Murrill state that in the treatment of these complaints, they have employed tar in two-grain doses, made into a pill, every three or four hours. From October to January, inclusive, its effects were watched on twenty-five patients, whose ages varied from thirty-four to seventy. All these patients had suffered several years from winter cough during the whole winter.

Each attack of the paroxysmal and violent cough lasted from two to ten minutes, recurring ten or twelve times in a day, and breaking their rest at night. Expectoration was abundant, frothy, and purulent. Breathing was short on exertion, but most could lie down at night without propping. The physical signs showed a variable amount of emphysema, with sonorous and sibilant rhonchus, and occasionally a little bubbling rhonchus at the base. These patients usually began to improve from the fourth to the seventh day; the improvement rapidly increased, and in about three weeks they were well enough to be discharged. The improvement was so decided that even those patients who, in previous years, had been confined to the house during the whole winter, returned to their work. On discontinuing the tar, relapses often occurred in a week or two, but on readministering the medicine relief was again obtained.

**Cystic Paralysis.**

Dr. J. Williamsky, (*Centralblatt fur Chirurgie,*) made some experiments with ergot upon the bladders of dogs and rabbits, in which he observed that immediate contraction of that organ ensued, and continued for several seconds. He afterward employed it in the form of injections of fluid extract in two cases of paralysis, with the following results: The first was a marked paralysis, occurring in a maiden lady of forty-six; division of a cicatrix between the posterior wall of the bladder and the neck of the uterus proved ineffectual. A small quantity of the fluid extract was then injected daily into the previously cleansed bladder, and a complete cure was effected in fourteen days. The second was a case of acute cystic catarrh with inability to completely empty the bladder. Three injections at intervals of twenty-four hours afforded entire relief.—*The Clinic.*

**Boils.**

DR. MADISON MARSH, of Port Hudson, La., says, in the *Medical and Surgical Reporter*, that boils and other analogous affections are treated by him with *sulphuric acid*, which he regards as almost a specific. He has used the acid with constant success for five and twenty years. "As soon as the patient applies for relief," he says, "I put an adult on elixir vitriol, twenty drops three times a day, in a glass of sweetened water, one hour before meals, previously smearing the teeth well with fresh butter or chewing a piece of fat pork, for a sure protection to the teeth. This is much better than sucking through a quill, as there is in this way regurgitation enough to throw the acid forward on the teeth. Using the butter or pork is a perfect protection, if the teeth are subsequently washed with a solution of bicarb. soda, a heaping teaspoonful to a glass of water. In the use of sulphuric acid in this way, the boil (or crop) then on hand will soon melt away, and there will be but one effort more to return before they will finally disappear, no more to reappear. The acid should be kept up in ten-drop doses for at least two weeks after the boils have disappeared. To assist in their local treatment, to effect a speedy cure and afford relief from pain and soreness, I apply a piece of common adhesive plaster, cut round, sufficiently large to cover the tumor to the extent of the areola, clipping the edges so that it will set smooth; or a little shoemaker's wax spread on a cloth will do just as well. I have made this application to saddle boils, and next day rode in the saddle quite comfortably, the boil progressing to maturity with great rapidity, with very little pain, and sometimes effecting an abortion at once.

"In this connection I have read in your *Periscope* a very interesting and scientific article on the sulphides of soda, potash, and calcium, as an antidote for all these ills of humanity. And now, right here let me suggest, perhaps the chemical play of affinities in *nature's* chemical laboratory may evolve the sulphides in the same way that chloral *amateurs* claim that hydrate chloral is metamorphosed in the blood to chloroform."

**Tincture of Capsicum in the Treatment of "Tippling."**

A correspondent of *Land and Water* throws out some suggestions to alleviate, if not cure, "tippling in private life." He says: Of course, as a rule, moral means, such as persuading or frightening the patient, are futile. Dr. Ringer, in an able article in the *British Medical Journal*, in 1874, advocated the use of capsicum, "given in doses of the tincture (ten drops), before meals, or whenever depression or craving for alcohol arises." It also induces sleep in early stages of delirium tremens. It obviates the morning vomiting, removes the sinking at the pit of the stomach, the intense craving for stimulants, and promotes appetite and digestion. This treatment I have tried with great success in several cases, and in one in particular, that of a young man, whom no one by any means in his power could possibly keep from tippling. Shut up the spirits, he had a key made on the quiet, while his wife was away for a day—of course he sent her. Take away his money, he would "tipple" on credit. He came under my care for bronchitis. I soon heard of the propensity, and tried Dr. Ringer's treatment. I began by giving him five drops of the tincture in a little syrup of orange peel, and some orange bitters, and increased the dose of capsicum to twelve drops. He rapidly improved, and at the end of a month he was quite another man. He was no longer to be seen in a half-muddled state, hanging about the low cabarets and taverns by himself, but every day walking out with his wife, and taking an interest in all that was going on. He left here for England about three months afterwards, and I have since heard that he takes to his bottle, (the capsicum bottle), whenever he feels inclined to indulge in any other sort of "tincture." Another case was that of a lady, over forty years of age, but not so successful as the one above cited. Of course, it is a great thing to wrap up the capsicum in a convenient vehicle, and the above, suggested to me by M. Dutertre, the well known pharmacien of our town, is, I think, as good a form as any.—*British Med. Jour.*

---

**How to Treat a Common Cold.**

Dr. J. M. Fothergill, in an article on this subject in the London *Practitioner*, remarks :—

Rarely is an impression made upon the pyrexia until the action of the skin is excited and the cooling effects of exhalation is attained. The administration of nauseant diaphoretics to attain these ends has been the rule among practitioners and housewives. The time-honored antimonial wine has scarcely yet yielded to its rival, ipecacuanha; nor, perhaps, is it desirable that it should. Their combination is good and to be recommended. In adults, iodide of potassium in guaiac mixture forms an excellent combination, especially when the cold is combined with rheumatic pains or tonsillitis. These internal remedies may be aided by external measures, such as warm baths. With children it is easy to wrap them up in a blanket wrung out of hot water, to inclose them so wrapped in a dry blanket, and to put them into bed. This may be repeated as required, and sufficiently aids the remedies given by the mouth. Measures for giving adults a warm bath in bed are now to be procured at little cost. After perspiration is once induced, there is a gradual fall of temperature; but the normal may not be reached for some days. There is a decided tendency to excessive heat-loss after the action of the skin has been established; even though the temperature indoors be above the normal. Experience has taught humanity to wrap up well when passing through a cold, especially when it is breaking. Ere the action of the skin is re-established, the impression of external cold is grateful, but afterward chills are readily experienced. The increase of blood in the heat-losing area permits of rapid heat-loss. When a cold is caught during the restorative period it is usually a fixed one, and not rarely serious illness is the consequence.

When the action of the skin is re-established, it not uncommonly happens that perspiration is profuse, even while the patients wrap up well to shield themselves from heat-loss. This is a troublesome stage in the history of a cold. Here mineral acids with vegetable tonics are indicated, and perhaps best of all, dilute phosphoric acid in cascarilla or cinchona. In the treatment of influenza, vegetable acids along with a bitter tonic should be given.

In the treatment of the bronchial affections which so commonly accompany an ordinary cold, it is not a matter of difference what expectorant remedy is selected. As long as the skin is dry, and the bronchial lining membrane tumid, and secretion arrested, ipecacuanha with acetate of ammonia is indicated; or a little antimony may be added with advantage. When the skin is once thrown into action and the bronchial secretion also established, then acids with syrup of squills are suitable measures. But it is not a successful plan to administer squills with acids until the skin is moist. When there is a tendency to the free action of the skin, this latter combination in full doses is a useful plan of treatment. Neither is the union of carbonate of ammonia and senega, in severe cases, indicated until the secretion alike of the bronchial lining membrane is thoroughly established.

---

**Typhoid Fever.**

A few cases have already been reported, but the case in question presented some features which made it one worthy of study. In the first place, the boy, æt. 21, and previously healthy, when admitted with this disease was at once placed upon the antipyretic treatment, by means of quinine. To this was added, after a few days, the hot pack. He was admitted Sept. 2, and on Sept. 17 his temperature was, at 1.30 P. M., 104¾°F., when a cold pack was ordered. The only symptom which indicated the intensity of the disease was the degree of body-heat which could be properly appreciated *only* by the use of the thermometer. The cold pack was used at 2.15 P. M., and the temperature fell to 103½°F., but it rose again, later in the afternoon, to 104°F., when the cold pack was again used, and the temperature fell half a degree. The packs were repeated during the night—were continued for fifteen minutes at each application, as a rule—but in the morning of the 18th the temperature was 104½°F., when the pack was used and it fell to 102½°F. It again rose to 103½°F., but the pack again brought it down to 102°F. In the afternoon of the same day the temperature, after a pack, fell to 102°F., then to 100°F., and finally it was brought down to 99°F. This was another remarkable feature, namely, the great decline of temperature so short a time before death, for the case terminated fatally on the morning of the 19th, the temperature having rapidly risen to 103°F. before the event occurred;

Digitalis was at once administered when the temperature fell to 99° F., but scarcely any effect was produced. The quinine was continued throughout the course of the disease. It was remarked that the fact should be borne in mind, that a method of treatment which apparently should succeed does sometimes fail, and that undue enthusiasm respecting any plan of treatment of disease should be received with a certain degree of reserve and scepticism. This patient succumbed, notwithstanding the temperature was brought down to nearly the normal standard.—*N. Y. Hosp. Rep.*

**Respiratory Percussion.**

In the *American Journal of Medical Sciences*, Dr. J. M. DaCosta describes this method of physical examination. By respiratory percussion is meant percussion after a full inspiration or a full expiration, the breath being suspended for a moment while the examination is being made.

The practical application of the respiratory method of percussion would appear to have a very wide range. In all chest affections, where percussion is used the modification may be employed with advantage. It often serves to clear up a diagnosis where the ordinary physical examination leaves the case doubtful, or where the usual signs tend to mislead. Thus, in bronchitis an abundant accumulation of secretion in the air-passages may obscure the breath sounds, and even give rise to a certain degree of dullness on percussion. But if the suspected region is percussed while the breath is held, after a full inspiration, the percussion note again becomes clear and the doubt is removed. On the other hand, should the dullness remain unchanged after inspiration, we may infer that the pulmonary tissue has suffered damage. In acute pneumonia, respiratory percussion may reveal commencing resolution before crepitation has appeared, and thus become available with respect to prognosis. In certain cases of organic heart-disease, where the symptoms lead to a suspicion of tubercular complication, this method of percussion, by causing the local congestion in the lungs to disappear, will greatly assist in the diagnosis. In pleurisy, with effusion, where the fluid is at the lower part of the lung, and some doubt exists as to whether it may not be a case of chronic pneumonia, by means of respiratory percussion the diagnosis may be rendered perfectly certain. If, after a forced respiration, the percussion developes a sharply defined line between the region of dullness below and resonance above, it is an effusion; while, if the dullness changes in part, or remains unchanged without being well defined, we may be sure that the lung is consolidated. Again, in those cases where in connection with an effusion in the pleura we get a bowing respiration at the back, with dullness on percussion, and it is a question whether pneumonia co-exists with the pleurisy. A full inspiration expands the lung tissue, if it be simply compressed or condensed, and the percussion sound becomes clear and resonant.

Obviously in phthisis, too, respiratory percussion may be made of great service in determining the existence of a deposit that is very slight in amount, or in ascertaining the degree of progress of the disease, by magnifying, as it were, the ordinary percussion signs, and by enabling us more accurately to define the limits of the disease. If both lungs are affected about equally, the presence of deposit will be denoted by the fact that respiratory percussion does not give an increased, but rather a diminished resonance, and after a forced expiration the dullness will be markedly increased. In the case of cavities the effect of a full inspiration upon the percussion sound is to change the tympanitic, cracked-pot, or amphoric notes to simple dullness, together with a higher pitch and a feeling of greater resistance.

In pneumothorax, respiratory percussion may be made available, it is claimed, to ascertain whether perforation between the lung and pleural cavity still remains pervious or not. If a full inspiration increases the resonance, the former is probably true; while if the resonance remains unchanged after inspiration, the aperture has closed. This, however, is not stated with complete certainty, and the necessity for further investigation is admitted. In emphysema the vesiculo-tympanitic note, elicited by ordinary percussion, is either not at all changed after forced inspiration, or but very slightly so, in case the emphysema is not marked.—*Med. Record.*

**Diarrhœa of Phthisis.**

Among the many remedies and combinations of remedies that have been used, the following prescriptions have obtained some reputation for good:

℞

Opium, ½ to 1 grain.

Bismuth subnit, gr. xv.

Ipecac, ½ grain. M.

Taken p. r. n.

℞

Nitrate of silver, ¼ gr. to ½ gr.

Opium, ½ gr. to 1 gr. M.

Ft. pil.

To be taken p. r. n.

℞

Strychnia sulp., $\frac{1}{24}$ gr.

Bismuth subnit, 15 to 20 gr. M.

Taken p. r. n.

